# Harnessing Gram‐negative bacteria for novel anti‐Gram‐negative antibiotics

**DOI:** 10.1111/1751-7915.70032

**Published:** 2024-11-02

**Authors:** Joy Birkelbach, Carsten E. Seyfert, Sebastian Walesch, Rolf Müller

**Affiliations:** ^1^ Helmholtz Institute for Pharmaceutical Research Saarland (HIPS) Helmholtz Centre for Infection Research (HZI) and Saarland University Department of Pharmacy Saarbrücken Germany; ^2^ German Centre for Infection Research (DZIF), Partner Site Hannover‐Braunschweig Braunschweig Germany

## Abstract

Natural products have proven themselves as a valuable resource for antibiotics. However, in view of increasing antimicrobial resistance, there is an urgent need for new, structurally diverse agents that have the potential to overcome resistance and treat Gram‐negative pathogens in particular. Historically, the search for new antibiotics was strongly focussed on the very successful Actinobacteria. On the other hand, other producer strains have been under‐sampled and their potential for the production of bioactive natural products has been underestimated. In this mini‐review, we highlight prominent examples of novel anti‐Gram negative natural products produced by Gram‐negative bacteria that are currently in lead optimisation or preclinical development. Furthermore, we will provide insights into the considerations and strategies behind the discovery of these agents and their putative applications.

## Graphical abstract

In this mini‐review, we highlight prominent examples of novel anti‐Gram negative natural products produced by isolated Gram‐negative bacteria that are currently in pre‐clinical development. Furthermore, we will provide insights into the considerations and strategies behind the discovery of these agents and their putative applications.

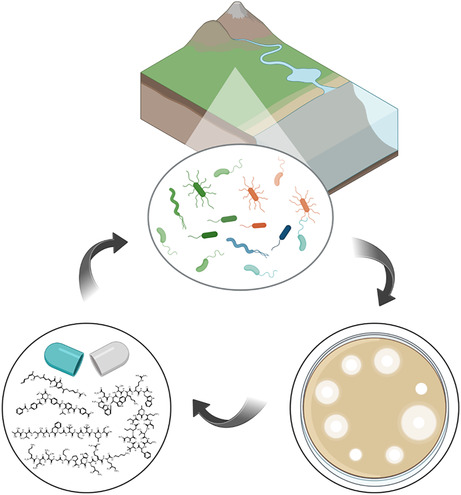

## INTRODUCTION – THE IMPORTANCE OF NATURAL PRODUCTS AS ANTIBIOTICS

Beginning with the development of salvarsan in the early 20th century (Ehrlich, 1913), small molecule antibiotics have become one of the pillars of modern medicine. These drugs gave humankind the ability to treat and cure a wide range of bacterial infections and paved the way for many modern medical procedures (Hutchings et al., 2019). However, this progress is jeopardised by the rise of antimicrobial resistances (AMR) against more and more classes of antibiotics, leading to increasing numbers of deaths that are associated with or directly attributed to AMR (Murray et al., 2022; O'Neil, 2014; Ventola, 2015). Therefore, the discovery and development of novel antibiotics with new chemical scaffolds and molecular targets are needed, in particular with activity against Gram‐negative bacteria (Walesch et al., 2022).

Although the first antibiotics were of synthetic nature (Ehrlich, 1913; Otten, 1986), the discoveries and subsequent use of penicillin and streptomycin shifted the focus of antibiotic discovery towards natural products from microbes (Abraham et al., 1941; Fleming, 1929; Waksman & Schatz, 1945; Walesch et al., 2022). The ongoing relevance of microbial natural products as antibiotics is attested by the fact that more than two thirds of antibiotics that were approved between 1981 and 2019 are natural products or derivatives thereof (Newman & Cragg, 2020). When taking into account antibiotics that are currently marketed in the United States for the systemic treatment of non‐mycobacterial infections, 18 out of 22 antibiotic classes are natural product‐based (Walesch et al., 2022). Compared to synthetic compounds, microbial natural products are believed optimised through evolution to facilitate microbial competition for nutrients and habitats. Therefore, their structural diversity and physico‐chemical properties are optimised to penetrate cell walls and selectively inhibit bacteria or other cells (Hutchings et al., 2019; Lakemeyer et al., 2018; Laraia & Waldmann, 2017; Wright, 2017).

**FIGURE 1:**
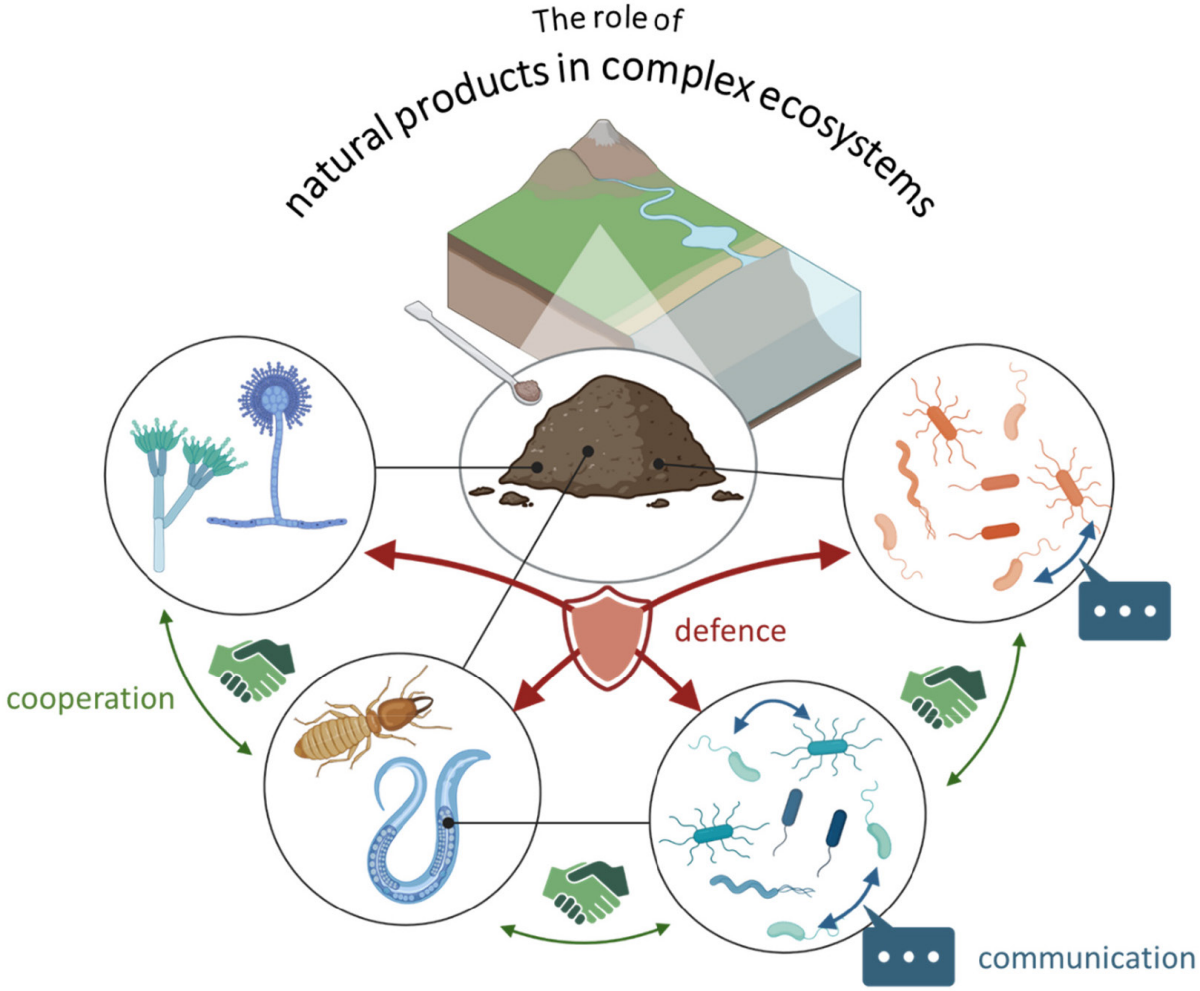
Microorganisms live in diverse habitats and ecosystems, as individuals, in competition or in symbiosis. Natural products are used as a means of communication, cooperation, inhibition, defence and predation. Exemplified by the cooperation of termites and fungi and of bacteria and nematodes, in defence of other microorganisms. Created in BioRender. Birkelbach, J. (2024) BioRender.com/x25z927

Bacteria deserve a special place among microbial natural products producers. According to the latest version of the MIBiG database, the structural and biosynthetic diversity of bacterial natural products exceeds that of the compounds produced by fungi (Terlouw et al., 2023). Furthermore, the organisation of the genes responsible for the production of natural products are mostly clustered in biosynthetic gene clusters (BGCs) facilitating genetic manipulations to study and optimise the production of such compounds (Bode & Müller, 2005). Bacteria can be isolated from diverse habitats ranging from marine to soil and from permafrost to desert environments. In these complex ecosystems, bacteria may live independently or as symbionts and their natural products play a key role fulfilling a broad range of biological functions, e.g., cooperation and communication, defence mechanisms and predation (Figure 1). A vast majority – more than 99% – of bacteria have not been cultivated, thus providing an immense potential to discover novel natural products (Crits‐Christoph et al., 2018; Locey & Lennon, 2016).

**FIGURE 2:**
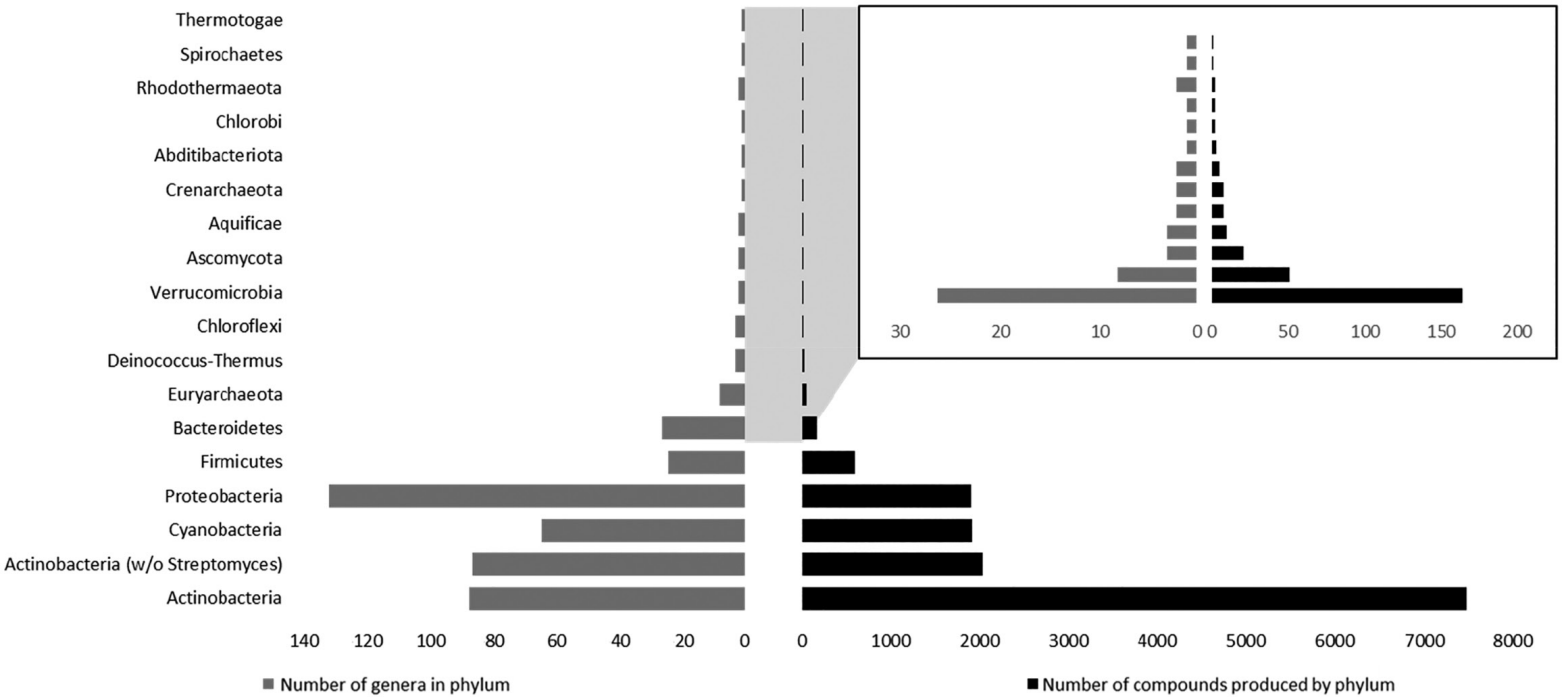
The number of bacterial natural products produced per phylum (left) versus the number of validly described genera per phylum (right) reported in the NPAtlas database (van Santen et al., 2022). Inset shows phyla that produce <200 reported natural products.

Hitherto, the majority of bacterial natural products listed in the NPAtlas database was discovered from Actinobacteria (Figure 2) (van Santen et al., 2022). Especially the genus *Streptomyces* was extremely fruitful for the discovery of clinically relevant antibiotics and anti‐cancer agents (Katz & Baltz, 2016; Newman & Cragg, 2020). However, the bias towards soil‐dwelling Actinobacteria and the resulting under‐sampling of other phyla underestimated the potential of other natural products producers (Hutchings et al., 2019). For example, the phyla Cyanobacteria, Proteobacteria, Firmicutes or Bacteriodetes are less explored than Actinobacteria and harbour more genera with less reported natural products (Figure 2) (van Santen et al., 2022; Walesch et al., 2022; Wright, 2017). In this mini‐review, we exemplarily highlight three different discovery approaches that were recently applied to generally underrepresented Gram‐negative producer strains, which yielded novel anti‐Gram negative antibiotics that are currently listed in preclinical development stages by the WHO, which comprise agents in lead optimisation, preclinical candidates applying Good Laboratory and Good Manufacturing Practices (GLP and GMP) and agents in Clinical Trial Application and Investigational New Drug‐enabling studies (CTA/IND) (Antimicrobial Resistance Division, 2024).

## MAIN – GRAM‐NEGATIVE BACTERIA PRODUCING ANTI‐GRAM‐NEGATIVE ANTIBIOTICS – APPROACHES/ HABITATS

### Predator approach

*Myxococcota* (or myxobacteria) are rod‐shaped Gram‐negative bacteria that grow in terrestrial and aquatic habitats all around the world (Mohr, 2018). They demonstrate a sophisticated and social life‐style including coordinated swarming on flat surfaces, cooperative predation of other microorganisms and the formation of multicellular fruiting bodies upon starvation (Munoz‐Dorado et al., 2016; Reichenbach, 1999). Furthermore, myxobacteria have the largest bacterial genomes with up to 16 Mbp (Han et al., 2013; Pal et al., 2021), harbouring a great potential to produce secondary metabolites (Garcia et al., 2024; Zaburannyi et al., 2016).

**FIGURE 3:**
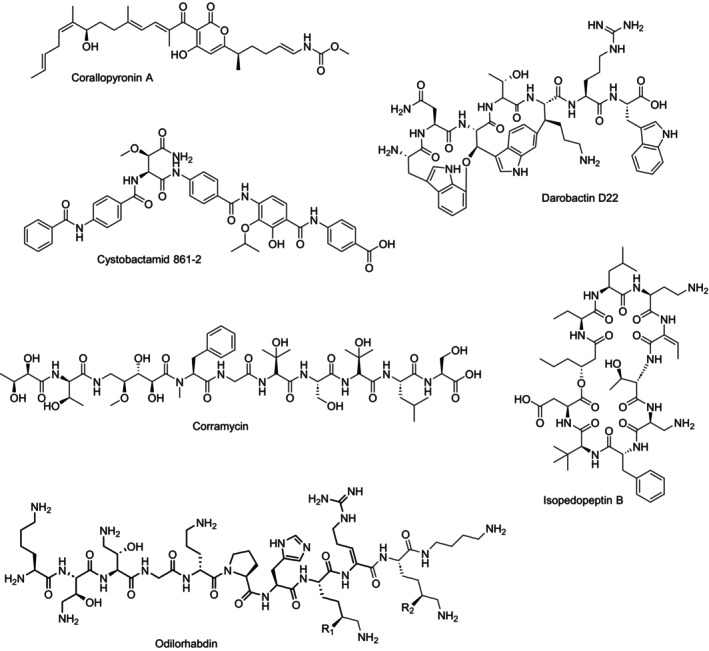
Chemical structures of corallopyronin A, cystobactamid 861–2, corramycin, isopedopeptin B, odilorhabdin and darobactin D22.

The potential value of myxobacteria as producers of antibiotics was first discussed in the middle of the past century, based on their ability to lyse other microbes (Oxford, 1947; Singh, 1947). Further research showed the ability of myxobacteria to lyse human pathogenic bacteria and to produce substances with antibacterial properties (Mathew & Dudani, 1955; Noren & Raper, 1962). Arguably, due to the comparably difficult (large scale) cultivation of most *Myxococcota* in the laboratory (Mohr, 2018), it took until the late 1970s before ambrucitin was isolated as the first natural product from a myxobacterium (Ringel et al., 1977). Since then, myxobacteria have proven themselves as a fruitful source of natural products with diverse chemical scaffolds displaying a wide range of biological activities (Herrmann et al., 2017). As of 2023, more than 800 natural products, belonging to ~170 chemical scaffolds were isolated from myxobacterial cultivation extracts (Wang et al., 2024). Among the many natural product scaffolds from myxobacteria with antibacterial activities, three compounds or compound classes are currently in preclinical development for their activities against Gram‐negative bacteria, corallopyronin A, cystobactamids and corramycins (Figure 3).

Although corallopyronin A (Figure 3) and its antibacterial activities against a range of Gram‐positive and ‐negative bacteria was already described in the 1980s (Irschik et al., 1985; Jansen et al., 1985), its development as antibiotic seemed unlikely, due to low production titres in the original producer *Corallococcus coralloides* and a poor yield in chemical synthesis (Krome et al., 2022). This changed with the implementation of a heterologous expression system of corallopyronin in *Myxococcus xanthus*, which increased the production of corallopyronin A greatly (Pogorevc et al., 2019; Sucipto et al., 2017). Corallopyronin A displays potent activity against Gram‐negative bacteria like *Neisseria ghonorrhoeae*, *Chlamydia* spp., *Rickettsia* spp. and *Wolbachia* spp. (Miethke et al., 2021). As a preclinical candidate it is currently developed for the treatment of filarial worm infections, by targeting their *Wolbachia* endosymbionts and has shown its efficacy in rodent infection models (Ehrens et al., 2022; Krome et al., 2022; Schiefer et al., 2020). The mode of action of corallopyronin A is the inhibition of the DNA‐dependent RNA polymerase (Mukhopadhyay et al., 2008). As its binding site at the ‘switch region’ of the enzyme is distinct from the binding sites of other RNA polymerase inhibitors, corallopyronin A does not show cross‐resistance with other antibiotics (Krome et al., 2022; Shima et al., 2018).

The antibacterial cystobactamids (Figure 3) were discovered in the cultivation extracts of *Cystobacter velatus* Cbv34 in the course of a screening campaign of a biodiverse collection of myxobacteria (Baumann et al., 2014; Herrmann et al., 2016). They are unusual peptides, featuring several *para*‐aminobenzoic acids and display activity against Gram‐positive and Gram‐negative bacteria (Baumann et al., 2014). The molecular target of cystobactamids, the bacterial type IIa topoisomerase was found through investigation of the self‐resistance mechanisms of the producing *Cystobacter* strain (Baumann et al., 2014). Obviously, cystobactamids target a different binding site in bacterial gyrases as the quinolone antibiotics, as they show a low to no cross‐resistance with this scaffold (Baumann et al., 2014; Hüttel et al., 2017). In the past years cystobactamids have progressed to the lead optimisation phase, as the implementation of a heterologous expression system and a total synthesis route have led to the development of derivatives with highly improved properties *in vitro* and *in vivo* (Elgaher et al., 2020; Groß et al., 2021; Moeller et al., 2019; Moreno et al., 2015; Testolin et al., 2020). Interestingly, the albicidins, originally isolated from the Gram‐negative bacterium *Xanthomonas albilineans*, have a similar scaffold and antibacterial activities to the cystobactamids (Cociancich et al., 2015). Due to their unique scaffold the cystobactamids and albicidins are currently in lead optimisation, which yielded derivatives with improved antibacterial properties and a better understanding of their modes of action and resistance mechanisms (Kleebauer et al., 2021; Michalczyk et al., 2023; Risch et al., 2024; Saathoff et al., 2023; Zborovsky et al., 2021).

Corramycins (Figure 3) are linear peptides with activity against *E. coli* that were first found in cultivation extracts of *Corallococcus coralloides* (Couturier et al., 2022). Lead optimisation by organic synthesis was used to develop a corramycin derivative with a more than 300‐fold increased activity against Gram‐negative bacteria, displaying promising activities against *K. pneumoniae* and *A. baumannii* (Renard et al., 2023). Inactivation of the warhead by phosphorylation was identified as a mechanism of self‐resistance in the producing myxobacteria (Adam et al., 2024). Although the mechanism of action of corramycins has not been elucidated yet, it can be assumed that it inhibits bacterial growth by a novel molecular target, as it shows no cross‐resistance with known antibiotic classes (Couturier et al., 2022; Renard et al., 2023). Moreover, corramycins have shown their *in vivo* efficacy in a range of rodent infection models (Couturier et al., 2022; Renard et al., 2023).

### Resistance‐based isolation approach

Antibiotic producing bacteria obviously require self‐resistance mechanisms to protect themselves against the toxicity of the natural product they produced to affect their opponent.

To exploit self‐resistance mechanisms, Thaker et al. successfully developed the resistance‐guided cultivation approach selecting for glycolipopetide‐resistant actinomycetes to screen for the production of new glycolipopeptides (Thaker et al., 2013, 2014). Bjerketorp et al. adapted and extended this approach on the screening of soil isolates selecting for environmental multi drug resistance (MDR) bacteria (Bjerketorp et al., 2021). Therefore, the combination of several antibiotics from different chemical classes was used to screen for natural product producers with multiple self‐resistance mechanisms and thus the capacity to produce several antibacterial compounds. Furthermore, it enabled the isolation of low‐abundant underexplored natural product producers present in soil‐samples (Bjerketorp et al., 2021), which would have proven difficult without the selection bias.

This approach led to the isolation of several MDR *Pedobacter* spp., which are Gram‐negative bacteria belonging to the phylum *Bacteroidota* and the family *Sphingobacteriaceae* (Bjerketorp et al., 2021). Nord et al. discovered the lipodepsipeptide isopedopeptin scaffold, which shows low micro‐molar activity against WHO top priority pathogens including colistin resistant strains (Nord et al., 2020). Membrane disruption is proposed as one mode of action (MoA) of isopedopeptins, which is corroborated by the structural similarity to bacterial lipopolysaccharide (LPS)‐binding pedopeptins (Hirota‐Takahata et al., 2014; Kozuma et al., 2014). However, the discrepancy with the MIC suggests additional MoAs. Currently, isopedopeptin B (ULT3, Figure 3) is in CTA/IND studies showing good anti‐Gram‐negative activity and acceptable cytotoxicity. Although it is expected to discover similar antibiotics compared to the ones the isolates are resistant to, the extended resistance‐based approach facilitated the isolation of underrepresented natural products producers, thereby the discovery of novel antibacterial compounds.

### Endosymbiont approach‐*Xenorhabdus* and *Photorhabdus* as a promising source of new anti‐Gram‐negatives

In addition to bacterial species that have long been studied for the discovery of novel antibiotics, such as *Actinomyces* or *Myxococcota* (Müller & Wink, 2014), entomopathogenic bacteria have become a focus of research interest due to their complex lifestyle (Chaston et al., 2011), which is mainly feasible due to the production of bioactive secondary metabolites that have a positive influence on persistence in the host (Cimen et al., 2022; Shi et al., 2022). In particular, studies have shown that the *γ*‐proteobacteria *Xenorhabdus* and *Photorhabdus* spp., that live in mutualistic symbiosis with nematodes, provide antibiotic candidates with promising anti‐Gram‐negative activity against difficult‐to‐treat pathogens (Chaston et al., 2011; Walesch et al., 2022). *Xenorhabdus* spp. primarily infect nematodes of the genus *Steinema*, whereas *Photorhabdus* spp. mainly infect *Heterorhabditis* spp. (Clarke, 2008; Forst, 2002; Poinar, 1966; Poinar & Thomas, 1967; Waterfield et al., 2001). Although they use functionally different approaches, both infect the intestinal tract of their host and are able to overcome or suppress the immune system of their hosts (Chaston et al., 2011; Clarke, 2008), or kill it by producing insecticides (Proschak et al., 2014; Sergeant et al., 2006). Furthermore, both are producing many antimicrobial compounds to prevent the growth of antagonistic microorganisms allowing the persistence in the intestinal tract of their nematodic host (Blackburn et al., 2016; Hu et al., 2006; Muangpat et al., 2017, 2020; Sajnaga & Kazimierczak, 2020; Wenski et al., 2020; Zhou et al., 2013). Recently, two promising novel antibacterial classes were discovered from these two endopathogenic bacteria: the odilorhabdins (Racine & Gualtieri, 2019) and the daropeptides (Ma et al., 2024), consisting of darobactins and dynobactin (Figure 3). Both classes exhibit strong anti‐Gram‐negative activity against, e.g., *Escherichia coli*, *Pseudomonas aeruginosa*, *Klebsiella pneumoniae* and *Acinetobacter baumannii* strains including clinical isolates (Chaston et al., 2011; Imai et al., 2019; Ma et al., 2024; Pantel et al., 2018).

#### Odilorhabdins

The discovery and development of odilorhabdins was largely driven by the start‐up Nosopharm (Racine & Gualtieri, 2019). In a comprehensive screening of various *Xenorhabdus* strains, the first odilorhabdins (Pantel et al., 2018) were isolated from *X. nematophila* using a traditional bioactivity‐guided approach, subsequently identifying the chemical structure and mode of action. Odilorhabdins represent a new class of broad‐spectrum antibiotics. These compounds target the 30S ribosomal subunit of Gram‐negative and Gram‐positive bacteria on a binding site not exploited by currently marketed antibiotics, thus they have a reduced risk of cross‐resistances (Pantel et al., 2018). Additionally, they conducted extensive derivatisation using chemical total synthesis to enhance antibacterial activity, particularly against *E. coli* and *K. pneumoniae* strains (Sarciaux et al., 2018). This led to the development of a frontrunner molecule, NOSO‐502 (Racine et al., 2018; Sarciaux et al., 2018), which is supposedly nearing clinical phase I studies for the treatment of critical urinary tract infections (UTIs) (Lanois‐Nouri et al., 2022; Präve et al., 2024; Racine et al., 2018; Racine & Gualtieri, 2019).

#### Darobactins

The first native darobactins were discovered after the bioactivity screening of *Photorhabdus* extracts (Imai et al., 2019). The bioactivity‐guided approach resulted in the isolation of darobactin A (DA) from *P. khanii*, a novel anti‐Gram‐negative agent that is a ribosomally synthesised and post‐translationally modified peptide (RiPP). Darobactins target the outer membrane protein BamA, part of the BamABCDE complex, preventing the incorporation and proper folding of outer membrane proteins into the cell membrane, thereby selectively killing Gram‐negative bacteria (Haysom et al., 2023; Imai et al., 2019; Kaur et al., 2021). Through binding on BamA, a novel antibacterial target, darobactins have a strongly reduced risk of cross‐resistance with currently used antibiotic classes.

Ongoing studies have focused on the heterologous production and total synthesis of native and artificial derivatives of darobactin A in *E. coli* by engineering the biosynthetic gene cluster (BGC) to produce optimised darobactin derivatives with modifications in the core peptide (Böhringer et al., 2021; Groß et al., 2021; Lin et al., 2022; Nesic et al., 2022; Seyfert et al., 2022; Wuisan et al., 2021). This has resulted in derivatives such as D22 (Figure 3) and D69, which exhibit up to 128‐fold enhanced *in vitro* anti‐Gram‐negative activity against pathogens classified as critical prioritised by the WHO, such as carbapenem‐resistant *A. baumannii* (CRAB), comparable to last‐resort antibiotics like colistin (Seyfert et al., 2022; Seyfert et al., 2023; World Health Organisation, 2018). Despite these advancements, both the total synthesis routes and the biotechnological production in alternative production hosts currently suffer from relatively low yields (Seyfert et al., 2023), which must be addressed to bring these highly promising agents into clinical development.

#### Outlook

The approaches described here, the predator approach, the resistance approach and the endosymbiont approach, led to promising anti‐Gram‐negative agents produced by Gram‐negative bacteria. Exemplary compounds such as cystobactamids, corramycins, corallopyoronin A, isopedopeptins, darobactins and odilorhabdins and their bioengineered or synthetic derivatives, respectively, are currently in development to prove their *in vivo* efficiency or have done so already (Couturier et al., 2022; Ehrens et al., 2022; Nord et al., 2020; Racine et al., 2018; Renard et al., 2023; Schiefer et al., 2020; Seyfert et al., 2023; Seyfert et al., 2023; Testolin et al., 2020). Those compounds, as well as other natural products in general, could be further optimised and their underlying biosynthesis investigated in more detail to allow for synthetic biology and evolution‐inspired bioengineering techniques (Bozhüyük et al., 2024; Präve et al., 2024). Moreover, novel bioinformatically guided tools could allow for the modification of the chemical structure, to, e.g., enhance target binding or alter the pharmaceutical properties (Ndagi et al., 2020; Wang et al., 2022). The discovery approaches described here, which aimed at identifying compounds with new target sites, unknown chemistry and exhibiting no cross‐resistances with marketed antibiotics, emphasise their potential for the development of novel molecules against Gram‐negative bacteria. Further approaches such as high‐throughput elicitor screening and the well‐established OSMAC approach enable the identification of additional antibacterial molecules. Those approaches can be used to investigate already cultivated but also uncultivated bacterial species, living in mostly underexplored habitats to discover novel chemistry to fight the AMR crisis (Bader et al., 2021; Claesen et al., 2020; Crits‐Christoph et al., 2018; Donia et al., 2014; Gavriilidou et al., 2022; Hegemann et al., 2023; Locey & Lennon, 2016; Nett et al., 2009; Nichols et al., 2010). As highlighted in Figure 2, e.g., *Pseudomonas*, *Burkholderia*, Cyanobacteria and Firmicutes species are further examples of underexplored yet promising natural products producers, which harbour the potential for the discovery of future antibiotic agents (Hegemann et al., 2023; van Santen et al., 2022; Walesch et al., 2022; Wright, 2017).

## AUTHOR CONTRIBUTIONS

**Joy Birkelbach:** Conceptualization; writing – original draft; writing – review and editing; visualization. **Carsten E. Seyfert:** Conceptualization; writing – original draft; writing – review and editing. **Sebastian Walesch:** Conceptualization; writing – original draft; writing – review and editing. **Rolf Müller:** Conceptualization; writing – review and editing; project administration; supervision.

## FUNDING INFORMATION

No funding information provided.

## CONFLICT OF INTEREST STATEMENT

C.E.S. and R.M. are inventors of the patent application WO 2022/175443 A1.

## References

[mbt270032-bib-0001] Abraham, E.P. , Chain, E. , Fletcher, C.M. , Gardner, A.D. , Heatley, N.G. , Jennings, M.A. et al. (1941) Further observations on penicillin. The Lancet, 238(6155), 177–189. 1541313

[mbt270032-bib-0002] Adam, S. , Fries, F. , Tesmar, A. , Rasheed, S. , Deckarm, S. , Sousa, C.F. et al. (2024) The peptide antibiotic Corramycin adopts a β‐hairpin‐like structure and is inactivated by the kinase ComG. Journal of the American Chemical Society, 146, 8981–8990. 38513269 10.1021/jacs.3c13208 PMC10996006

[mbt270032-bib-0003] Antimicrobial Resistance Division . (2024) 2023 Antibacterial agents in clinical and preclinical development: an overview and analysis. Geneva: World Health Organization.

[mbt270032-bib-0004] Bader, C.D. , Haack, P.A. , Panter, F. , Krug, D. & Müller, R. (2021) Expanding the scope of detectable microbial natural products by complementary analytical methods and cultivation systems. Journal of Natural Products, 84(2), 268–277. 33449690 10.1021/acs.jnatprod.0c00942

[mbt270032-bib-0005] Baumann, S. , Herrmann, J. , Raju, R. , Steinmetz, H. , Mohr, K.I. , Hüttel, S. et al. (2014) Cystobactamids: myxobacterial topoisomerase inhibitors exhibiting potent antibacterial activity. Angewandte Chemie, International Edition, 53(52), 14605–14609. 25510965 10.1002/anie.201409964

[mbt270032-bib-0006] Bjerketorp, J. , Levenfors, J.J. , Nord, C. , Guss, B. , Öberg, B. & Broberg, A. (2021) Selective isolation of multidrug‐resistant Pedobacter spp., producers of novel antibacterial peptides. Frontiers in Microbiology, 12, 642829. 33717041 10.3389/fmicb.2021.642829 PMC7947920

[mbt270032-bib-0007] Blackburn, D. , Wood, P.L. , Burk, T.J. , Crawford, B. , Wright, S.M. & Adams, B.J. (2016) Evolution of virulence in Photorhabdus spp., entomopathogenic nematode symbionts. Systematic and Applied Microbiology, 39(3), 173–179. 27020955 10.1016/j.syapm.2016.02.003

[mbt270032-bib-0008] Bode, H.B. & Müller, R. (2005) The impact of bacterial genomics on natural product research. Angewandte Chemie, International Edition, 44(42), 6828–6846. 16249991 10.1002/anie.200501080

[mbt270032-bib-0009] Böhringer, N. , Green, R. , Liu, Y. , Mettal, U. , Marner, M. , Modaresi, S.M. et al. (2021) Mutasynthetic production and antimicrobial characterization of Darobactin analogs. Microbiology Spectrum, 9(3), e0153521. 34937193 10.1128/spectrum.01535-21 PMC8694152

[mbt270032-bib-0010] Bozhüyük, K.A.J. , Präve, L. , Kegler, C. , Schenk, L. , Kaiser, S. , Schelhas, C. et al. (2024) Evolution‐inspired engineering of nonribosomal peptide synthetases. Science, 383(6689), eadg4320. 38513038 10.1126/science.adg4320

[mbt270032-bib-0011] Chaston, J.M. , Suen, G. , Tucker, S.L. , Andersen, A.W. , Bhasin, A. , Bode, E. et al. (2011) The entomopathogenic bacterial endosymbionts Xenorhabdus and Photorhabdus: convergent lifestyles from divergent genomes. PLoS One, 6(11), e27909. 22125637 10.1371/journal.pone.0027909 PMC3220699

[mbt270032-bib-0012] Cimen, H. , Touray, M. , Gulsen, S.H. & Hazir, S. (2022) Natural products from Photorhabdus and Xenorhabdus: mechanisms and impacts. Applied Microbiology and Biotechnology, 106(12), 4387–4399. 35723692 10.1007/s00253-022-12023-9

[mbt270032-bib-0013] Claesen, J. , Spagnolo, J.B. , Ramos, S.F. , Kurita, K.L. , Byrd, A.L. , Aksenov, A.A. et al. (2020) A Cutibacterium acnes antibiotic modulates human skin microbiota composition in hair follicles. Science Translational Medicine, 12(570), eaay5445. 33208503 10.1126/scitranslmed.aay5445 PMC8478231

[mbt270032-bib-0014] Clarke, D.J. (2008) Photorhabdus: a model for the analysis of pathogenicity and mutualism. Cellular Microbiology, 10(11), 2159–2167. 18647173 10.1111/j.1462-5822.2008.01209.x

[mbt270032-bib-0015] Cociancich, S. , Pesic, A. , Petras, D. , Uhlmann, S. , Kretz, J. , Schubert, V. et al. (2015) The gyrase inhibitor albicidin consists of *p*‐aminobenzoic acids and cyanoalanine. Nature Chemical Biology, 11(3), 195–197. 25599532 10.1038/nchembio.1734

[mbt270032-bib-0016] Couturier, C. , Groß, S. , von Tesmar, A. , Hoffmann, J. , Deckarm, S. , Fievet, A. et al. (2022) Structure elucidation, total synthesis, antibacterial in vivo efficacy and biosynthesis proposal of Myxobacterial Corramycin. Angewandte Chemie, International Edition in English, 61, e202210747. Available from: 10.1002/anie.202210747 PMC10099666 36197755

[mbt270032-bib-0017] Crits‐Christoph, A. , Diamond, S. , Butterfield, C.N. , Thomas, B.C. & Banfield, J.F. (2018) Novel soil bacteria possess diverse genes for secondary metabolite biosynthesis. Nature, 558(7710), 440–444. 29899444 10.1038/s41586-018-0207-y

[mbt270032-bib-0018] Donia, M.S. , Cimermancic, P. , Schulze, C.J. , Wieland Brown, L.C. , Martin, J. , Mitreva, M. et al. (2014) A systematic analysis of biosynthetic gene clusters in the human microbiome reveals a common family of antibiotics. Cell, 158(6), 1402–1414. 25215495 10.1016/j.cell.2014.08.032 PMC4164201

[mbt270032-bib-0019] Ehrens, A. , Schiefer, A. , Krome, A.K. , Becker, T. , Rox, K. , Neufeld, H. et al. (2022) Pharmacology and early ADMET data of corallopyronin a, a natural product with macrofilaricidal anti‐wolbachial activity in filarial nematodes. Frontiers in Tropical Diseases, 39(9), 1705–1720.

[mbt270032-bib-0020] Ehrlich, P. (1913) Address in pathology, ON CHEMIOTHERAPY: delivered before the seventeenth international congress of medicine. British Medical Journal, 2(2746), 353–359. 20766753 10.1136/bmj.2.2746.353 PMC2345634

[mbt270032-bib-0021] Elgaher, W.A.M. , Hamed, M.M. , Baumann, S. , Herrmann, J. , Siebenbürger, L. , Krull, J. et al. (2020) Cystobactamid 507: concise synthesis, mode of action and optimization toward more potent antibiotics. Chemistry – A European Journal, 26, 7219–7225. 31984562 10.1002/chem.202000117 PMC7317206

[mbt270032-bib-0022] Fleming, A. (1929) On the antibacterial action of cultures of a Penicillium, with special reference to their use in the isolation of *B. influenzæ* . British Journal of Experimental Pathology, 10(3), 226–236.

[mbt270032-bib-0023] Forst, S. (2002) Bacteria‐nematode symbiosis. In: Entomopathogenic nematology, p. 57. Wallingford: CABI Publishing UK.

[mbt270032-bib-0024] Garcia, R. , Popoff, A. , Bader, C.D. , Löhr, J. , Walesch, S. , Walt, C. et al. (2024) Discovery of the Pendulisporaceae: an extremotolerant myxobacterial family with distinct sporulation behavior and prolific specialized metabolism. Chem, 10, 1–20.

[mbt270032-bib-0025] Gavriilidou, A. , Kautsar, S.A. , Zaburannyi, N. , Krug, D. , Müller, R. , Medema, M.H. et al. (2022) Compendium of specialized metabolite biosynthetic diversity encoded in bacterial genomes. Nature Microbiology, 7(5), 726–735. 10.1038/s41564-022-01110-2 35505244

[mbt270032-bib-0026] Groß, S. , Panter, F. , Pogorevc, D. , Seyfert, C.E. , Deckarm, S. , Bader, C.D. et al. (2021) Improved broad‐spectrum antibiotics against gram‐negative pathogens via darobactin biosynthetic pathway engineering. Chemical Science, 12(35), 11882–11893. 34659729 10.1039/d1sc02725e PMC8442675

[mbt270032-bib-0027] Groß, S. , Schnell, B. , Haack, P.A. , Auerbach, D. & Müller, R. (2021) In vivo and in vitro reconstitution of unique key steps in cystobactamid antibiotic biosynthesis. Nature Communications, 12(1), 1696. 10.1038/s41467-021-21848-3 PMC7966384 33727542

[mbt270032-bib-0028] Han, K. , Li, Z.‐F. , Peng, R. , Zhu, L.‐P. , Zhou, T. , Wang, L. et al. (2013) Extraordinary expansion of a *Sorangium cellulosum* genome from an alkaline milieu. Scientific Reports, 3, 2101. 23812535 10.1038/srep02101 PMC3696898

[mbt270032-bib-0029] Haysom, S.F. , Machin, J. , Whitehouse, J.M. , Horne, J.E. , Fenn, K. , Ma, Y. et al. (2023) Darobactin B stabilises a lateral‐closed conformation of the BAM complex in *E. coli* cells. Angewandte Chemie, 62(34), e202218783. 37162386 10.1002/anie.202218783 PMC10952311

[mbt270032-bib-0030] Hegemann, J.D. , Birkelbach, J. , Walesch, S. & Müller, R. (2023) Current developments in antibiotic discovery: global microbial diversity as a source for evolutionary optimized anti‐bacterials: global microbial diversity as a source for evolutionary optimized anti‐bacterials. EMBO Reports, 24(1), e56184. 36541849 10.15252/embr.202256184 PMC9827545

[mbt270032-bib-0031] Herrmann, J. , Fayad, A.A. & Müller, R. (2017) Natural products from myxobacteria: novel metabolites and bioactivities. Natural Product Reports, 34(2), 135–160. 27907217 10.1039/c6np00106h

[mbt270032-bib-0032] Herrmann, J. , Lukezic, T. , Kling, A. , Baumann, S. , Hüttel, S. , Petkovic, H. et al. (2016) Strategies for the discovery and development of new antibiotics from natural products: three case studies. Current Topics in Microbiology and Immunology, 398, 339–363. 27738913 10.1007/82_2016_498

[mbt270032-bib-0033] Hirota‐Takahata, Y. , Kozuma, S. , Kuraya, N. , Fukuda, D. , Nakajima, M. & Ando, O. (2014) Pedopeptins, novel inhibitors of LPS: taxonomy of producing organism, fermentation, isolation, physicochemical properties and structural elucidation. Journal of Antibiotics (Tokyo), 67(3), 243–251. 10.1038/ja.2013.122 24301185

[mbt270032-bib-0034] Hu, K.J. , Li, J.X. , Li, B. , Webster, J.M. & Chen, G.H. (2006) A novel antimicrobial epoxide isolated from larval galleria mellonella infected by the nematode symbiont, *Photorhabdus luminescens* (Enterobacteriaceae). Bioorganic & Medicinal Chemistry, 14(13), 4677–4681. 16644226 10.1016/j.bmc.2006.01.025

[mbt270032-bib-0035] Hutchings, M.I. , Truman, A.W. & Wilkinson, B. (2019) Antibiotics: past, present and future. Current Opinion in Microbiology, 51, 72–80. 31733401 10.1016/j.mib.2019.10.008

[mbt270032-bib-0036] Hüttel, S. , Testolin, G. , Herrmann, J. , Planke, T. , Gille, F. , Moreno, M. et al. (2017) Discovery and total synthesis of natural Cystobactamid derivatives with superior activity against gram‐negative pathogens. Angewandte Chemie, International Edition, 56(41), 12760–12764. 28730677 10.1002/anie.201705913

[mbt270032-bib-0037] Imai, Y. , Meyer, K.J. , Iinishi, A. , Favre‐Godal, Q. , Green, R. , Manuse, S. et al. (2019) A new antibiotic selectively kills gram‐negative pathogens. Nature, 576(7787), 459–464. 31747680 10.1038/s41586-019-1791-1 PMC7188312

[mbt270032-bib-0038] Irschik, H. , Jansen, R. , Höfle, G. , Gerth, K. & Reichenbach, H. (1985) The corallopyronins, new inhibitors of bacterial RNA synthesis from Myxobacteria. The Journal of Antibiotics, 38(2), 145–152. 2581926 10.7164/antibiotics.38.145

[mbt270032-bib-0039] Jansen, R. , Höfle, G. , Irschik, H. & Reichenbach, H. (1985) Antibiotika aus Gleitenden Bakterien, XXIV. Corallopyronin a, B und C – drei neue Antibiotika aus *Corallococcus coralloides* cc c127 (Myxobacterales). Liebigs Annalen der Chemie, 1985(4), 822–836.

[mbt270032-bib-0040] Katz, L. & Baltz, R.H. (2016) Natural product discovery: past, present, and future. Journal of Industrial Microbiology & Biotechnology, 43(2–3), 155–176. 26739136 10.1007/s10295-015-1723-5

[mbt270032-bib-0041] Kaur, H. , Jakob, R.P. , Marzinek, J.K. , Green, R. , Imai, Y. , Bolla, J.R. et al. (2021) The antibiotic darobactin mimics a β‐strand to inhibit outer membrane insertase. Nature, 593, 125–129. 33854236 10.1038/s41586-021-03455-w

[mbt270032-bib-0042] Kleebauer, L. , Zborovsky, L. , Hommernick, K. , Seidel, M. , Weston, J.B. & Süssmuth, R.D. (2021) Overcoming AlbD protease resistance and improving potency: synthesis and bioactivity of antibacterial Albicidin analogues with amide Bond Isosteres. Organic Letters, 23(18), 7023–7027. 34398605 10.1021/acs.orglett.1c02312

[mbt270032-bib-0043] Kozuma, S. , Hirota‐Takahata, Y. , Fukuda, D. , Kuraya, N. , Nakajima, M. & Ando, O. (2014) Screening and biological activities of pedopeptins, novel inhibitors of LPS produced by soil bacteria. Journal of Antibiotics, 67(3), 237–242. 24281661 10.1038/ja.2013.121

[mbt270032-bib-0044] Krome, A.K. , Becker, T. , Kehraus, S. , Schiefer, A. , Gütschow, M. , Chaverra‐Muñoz, L. et al. (2022) Corallopyronin A: antimicrobial discovery to preclinical development. Natural Product Reports, 39(9), 1705–1720. 35730490 10.1039/d2np00012a

[mbt270032-bib-0045] Lakemeyer, M. , Zhao, W. , Mandl, F.A. , Hammann, P. & Sieber, S.A. (2018) Thinking outside the box‐novel Antibacterials to tackle the resistance crisis. Angewandte Chemie, 57(44), 14440–14475. 29939462 10.1002/anie.201804971

[mbt270032-bib-0046] Lanois‐Nouri, A. , Pantel, L. , Fu, J. , Houard, J. , Ogier, J.‐C. , Polikanov, Y.S. et al. (2022) The Odilorhabdin antibiotic biosynthetic cluster and acetyltransferase self‐resistance locus are niche and species specific. MBio, 13, e0282621. 35012352 10.1128/mbio.02826-21 PMC8749412

[mbt270032-bib-0047] Laraia, L. & Waldmann, H. (2017) Natural product inspired compound collections: evolutionary principle, chemical synthesis, phenotypic screening, and target identification. Drug Discovery Today: Technologies, 23, 75–82. 28647090 10.1016/j.ddtec.2017.03.003

[mbt270032-bib-0048] Lin, Y.‐C. , Schneider, F. , Eberle, K.J. , Chiodi, D. , Nakamura, H. , Reisberg, S.H. et al. (2022) Atroposelective total synthesis of Darobactin a. Journal of the American Chemical Society, 144(32), 14458–14462. 35926121 10.1021/jacs.2c05892 PMC9829381

[mbt270032-bib-0049] Locey, K.J. & Lennon, J.T. (2016) Scaling laws predict global microbial diversity. Proceedings of the National Academy of Sciences, USA, 113(21), 5970–5975. 10.1073/pnas.1521291113 PMC4889364 27140646

[mbt270032-bib-0050] Ma, S. , Guo, S. , Ding, W. & Zhang, Q. (2024) Daropeptide natural products. Exploration of Drug Science, 2, 190–202.

[mbt270032-bib-0051] Mathew, S. & Dudani, A. (1955) Lysis of human pathogenic bacteria by myxobacteria. Nature, 175(4446), 125. 13235825 10.1038/175125a0

[mbt270032-bib-0052] Michalczyk, E. , Hommernick, K. , Behroz, I. , Kulike, M. , Pakosz‐Stępień, Z. , Mazurek, L. et al. (2023) Molecular mechanism of topoisomerase poisoning by the peptide antibiotic albicidin. Nature Catalysis, 6(1), 52–67. 10.1038/s41929-022-00904-1 PMC9886550 36741192

[mbt270032-bib-0053] Miethke, M. , Pieroni, M. , Weber, T. , Brönstrup, M. , Hammann, P. , Halby, L. et al. (2021) Towards the sustainable discovery and development of new antibiotics. Nature Reviews Chemistry, 5, 726–749. 10.1038/s41570-021-00313-1 PMC8374425 34426795

[mbt270032-bib-0054] Moeller, M. , Norris, M.D. , Planke, T. , Cirnski, K. , Herrmann, J. , Müller, R. et al. (2019) Scalable syntheses of Methoxyaspartate and preparation of the antibiotic Cystobactamid 861‐2 and highly potent derivatives. Organic Letters, 21(20), 8369–8372. 31599597 10.1021/acs.orglett.9b03143

[mbt270032-bib-0055] Mohr, K.I. (2018) Diversity of Myxobacteria‐we only see the tip of the iceberg. Microorganisms, 6(3), 84. 30103481 10.3390/microorganisms6030084 PMC6164225

[mbt270032-bib-0056] Moreno, M. , Elgaher, W. , Herrmann, J. , Schläger, N. , Hamed, M. , Baumann, S. et al. (2015) Synthesis and biological evaluation of cystobactamid 507: a bacterial topoisomerase inhibitor from *Cystobacter* sp. Synlett, 26(9), 1175–1178.

[mbt270032-bib-0057] Muangpat, P. , Suwannaroj, M. , Yimthin, T. , Fukruksa, C. , Sitthisak, S. , Chantratita, N. et al. (2020) Antibacterial activity of Xenorhabdus and Photorhabdus isolated from entomopathogenic nematodes against antibiotic‐resistant bacteria. PLoS One, 15(6), e0234129. 32502188 10.1371/journal.pone.0234129 PMC7274414

[mbt270032-bib-0058] Muangpat, P. , Yooyangket, T. , Fukruksa, C. , Suwannaroj, M. , Yimthin, T. , Sitthisak, S. et al. (2017) Screening of the antimicrobial activity against drug resistant bacteria of Photorhabdus and Xenorhabdus associated with Entomopathogenic nematodes from Mae Wong National Park, Thailand. Frontiers in Microbiology, 8, 1142. 28702004 10.3389/fmicb.2017.01142 PMC5487437

[mbt270032-bib-0059] Mukhopadhyay, J. , Das, K. , Ismail, S. , Koppstein, D. , Jang, M. , Hudson, B. et al. (2008) The RNA polymerase “switch region” is a target of inhibitors. Cell, 135, 295–307. 18957204 10.1016/j.cell.2008.09.033 PMC2580802

[mbt270032-bib-0060] Müller, R. & Wink, J. (2014) Future potential for anti‐infectives from bacteria – how to exploit biodiversity and genomic potential. International Journal of Medical Microbiology, 304(1), 3–13. 24119567 10.1016/j.ijmm.2013.09.004

[mbt270032-bib-0061] Munoz‐Dorado, J. , Marcos‐Torres, F.J. , Garcia‐Bravo, E. , Moraleda‐Munoz, A. & Perez, J. (2016) Myxobacteria: moving, killing, feeding, and surviving together. Frontiers in Microbiology, 7, 781. 27303375 10.3389/fmicb.2016.00781 PMC4880591

[mbt270032-bib-0062] Murray, C.J.L. , Ikuta, K.S. , Sharara, F. , Swetschinski, L. , Robles Aguilar, G. , Gray, A. et al. (2022) Global burden of bacterial antimicrobial resistance in 2019: a systematic analysis. Lancet, 399(10325), 629–655. 35065702 10.1016/S0140-6736(21)02724-0 PMC8841637

[mbt270032-bib-0063] Ndagi, U. , Falaki, A.A. , Abdullahi, M. , Lawal, M.M. & Soliman, M.E. (2020) Antibiotic resistance: bioinformatics‐based understanding as a functional strategy for drug design. RSC Advances, 10(31), 18451–18468. 35685616 10.1039/d0ra01484b PMC9122625

[mbt270032-bib-0064] Nesic, M. , Ryffel, D.B. , Maturano, J. , Shevlin, M. , Pollack, S.R. , Gauthier, D.R. et al. (2022) Total synthesis of Darobactin a. Journal of the American Chemical Society, 144(31), 14026–14030. 35900216 10.1021/jacs.2c05891

[mbt270032-bib-0065] Nett, M. , Ikeda, H. & Moore, B.S. (2009) Genomic basis for natural product biosynthetic diversity in the actinomycetes. Natural Product Reports, 26(11), 1362–1384. 19844637 10.1039/b817069j PMC3063060

[mbt270032-bib-0066] Newman, D.J. & Cragg, G.M. (2020) Natural products as sources of new drugs over the nearly four decades from 01/1981 to 09/2019. Journal of Natural Products, 83(3), 770–803. 32162523 10.1021/acs.jnatprod.9b01285

[mbt270032-bib-0067] Nichols, D. , Cahoon, N. , Trakhtenberg, E.M. , Pham, L. , Mehta, A. , Belanger, A. et al. (2010) Use of Ichip for high‐throughput *in situ* cultivation of “uncultivable” microbial species. Applied and Environmental Microbiology, 76(8), 2445–2450. 20173072 10.1128/AEM.01754-09 PMC2849220

[mbt270032-bib-0068] Nord, C. , Bjerketorp, J. , Levenfors, J.J. , Cao, S. , Strömstedt, A.A. , Guss, B. et al. (2020) Isopedopeptins A‐H: cationic cyclic Lipodepsipeptides from *Pedobacter cryoconitis* UP508 targeting WHO top‐priority Carbapenem‐resistant bacteria. ACS Chemical Biology, 15(11), 2937–2944. 33054165 10.1021/acschembio.0c00568 PMC7684578

[mbt270032-bib-0069] Noren, B. & Raper, K. (1962) Antibiotic activity of myxobacteria in relation to their bacteriolytic capacity. Journal of Bacteriology, 84(1), 157–162. 14480333 10.1128/jb.84.1.157-162.1962 PMC277787

[mbt270032-bib-0070] O'Neil, J. (2014) Antimicrobial resistance: tackling a crisis for the health and wealth of nations.

[mbt270032-bib-0071] Otten, H. (1986) Domagk and the development of the sulphonamides. The Journal of Antimicrobial Chemotherapy, 17(6), 689–696. 3525495 10.1093/jac/17.6.689

[mbt270032-bib-0072] Oxford, A. (1947) Observations concerning the growth and metabolic activities of myxococci in a simple protein‐free liquid medium. Journal of Bacteriology, 53, 129–138. 16561256 10.1128/jb.53.2.129-138.1947 PMC518287

[mbt270032-bib-0073] Pal, S. , Sharma, G. & Subramanian, S. (2021) Complete genome sequence and identification of polyunsaturated fatty acid biosynthesis genes of the myxobacterium *Minicystis rosea* DSM 24000T. BMC Genetics, 22(1), 655. 10.1186/s12864-021-07955-x PMC8436480 34511070

[mbt270032-bib-0074] Pantel, L. , Florin, T. , Dobosz‐Bartoszek, M. , Racine, E. , Sarciaux, M. , Serri, M. et al. (2018) Odilorhabdins, antibacterial agents that cause miscoding by binding at a new ribosomal site. Molecular Cell, 70(1), 83–94.e7. 29625040 10.1016/j.molcel.2018.03.001

[mbt270032-bib-0075] Pogorevc, D. , Panter, F. , Schillinger, C. , Jansen, R. , Wenzel, S.C. & Müller, R. (2019) Production optimization and biosynthesis revision of corallopyronin a, a potent anti‐filarial antibiotic. Metabolic Engineering, 55, 201–211. 31340171 10.1016/j.ymben.2019.07.010

[mbt270032-bib-0076] Poinar, G.O. (1966) The presence of Achromobacter Nematophilus in the infective stage of a Neoaplectana Sp. (Steinernematidae: Nematoda). Nematology, 12(1), 105–108.

[mbt270032-bib-0077] Poinar, G.O. & Thomas, G.M. (1967) The nature of *Achromobacter nematophilus* as an insect pathogen. Journal of Invertebrate Pathology, 9(4), 510–514.

[mbt270032-bib-0078] Präve, L. , Seyfert, C.E. , Bozhüyük, K.A.J. , Racine, E. , Müller, R. & Bode, H.B. (2024) Investigation of the Odilorhabdin biosynthetic gene cluster using NRPS engineering. Angewandte Chemie, 63(33), e202406389. 38801753 10.1002/anie.202406389

[mbt270032-bib-0079] Proschak, A. , Zhou, Q. , Schöner, T. , Thanwisai, A. , Kresovic, D. , Dowling, A. et al. (2014) Biosynthesis of the insecticidal xenocyloins in *Xenorhabdus bovienii* . Chembiochem, 15(3), 369–372. 24488732 10.1002/cbic.201300694

[mbt270032-bib-0080] Racine, E. & Gualtieri, M. (2019) From Worms to drug candidate: the story of Odilorhabdins, a new class of antimicrobial agents. Frontiers in Microbiology, 10, 2893. 31921069 10.3389/fmicb.2019.02893 PMC6930155

[mbt270032-bib-0081] Racine, E. , Nordmann, P. , Pantel, L. , Sarciaux, M. , Serri, M. , Houard, J. et al. (2018) In vitro and in vivo characterization of NOSO‐502, a novel inhibitor of bacterial translation. Antimicrobial Agents and Chemotherapy, 62(9), e01016. 29987155 10.1128/AAC.01016-18 PMC6125496

[mbt270032-bib-0082] Reichenbach, H. (1999) The ecology of the myxobacteria. Environmental Microbiology, 1(1), 15–21. 11207714 10.1046/j.1462-2920.1999.00016.x

[mbt270032-bib-0083] Renard, S. , Versluys, S. , Taillier, T. , Dubarry, N. , Leroi‐Geissler, C. , Rey, A. et al. (2023) Optimization of the antibacterial Spectrum and the Developability profile of the novel‐class natural product Corramycin. Journal of Medicinal Chemistry, 66(24), 16869–16887. 38088830 10.1021/acs.jmedchem.3c01564

[mbt270032-bib-0084] Ringel, S.M. , Greenough, R.C. , Roemer, S. , Connor, D. , Gutt, A.L. , Blair, B. et al. (1977) Ambruticin (W7783), a new antifungal antibiotic. The Journal of Antibiotics, 30(5), 371–375. 407203 10.7164/antibiotics.30.371

[mbt270032-bib-0085] Risch, T. , Kolling, D. , Mostert, D. , Seedorf, T. , Heimann, D. , Kohnhäuser, D. et al. (2024) Point mutations in the ygiV promoter region lead to cystobactamid resistance and reduced virulence in *E. coli* . NPJ Antimicrobial Resistance. doi: 10.1038/s44259-024-00050-7 PMC11721078 39843645

[mbt270032-bib-0086] Saathoff, M. , Kosol, S. , Semmler, T. , Tedin, K. , Dimos, N. , Kupke, J. et al. (2023) Gene amplifications cause high‐level resistance against albicidin in gram‐negative bacteria. PLoS Biology, 21(8), e3002186. 37561817 10.1371/journal.pbio.3002186 PMC10414762

[mbt270032-bib-0087] Sajnaga, E. & Kazimierczak, W. (2020) Evolution and taxonomy of nematode‐associated entomopathogenic bacteria of the genera Xenorhabdus and Photorhabdus: an overview. Symbiosis, 80(1), 1–13.

[mbt270032-bib-0088] Sarciaux, M. , Pantel, L. , Midrier, C. , Serri, M. , Gerber, C. , Marcia de Figueiredo, R. et al. (2018) Total synthesis and structure‐activity relationships study of Odilorhabdins, a new class of peptides showing potent antibacterial activity. Journal of Medicinal and Pharmaceutical Chemistry, 61(17), 7814–7826. 10.1021/acs.jmedchem.8b00790 30086230

[mbt270032-bib-0089] Schiefer, A. , Hübner, M.P. , Krome, A. , Lämmer, C. , Ehrens, A. , Aden, T. et al. (2020) Corallopyronin a for short‐course anti‐wolbachial, macrofilaricidal treatment of filarial infections. PLoS Neglected Tropical Diseases, 14(12), e0008930. 33284808 10.1371/journal.pntd.0008930 PMC7746275

[mbt270032-bib-0090] Sergeant, M. , Baxter, L. , Jarrett, P. , Shaw, E. , Ousley, M. , Winstanley, C. et al. (2006) Identification, typing, and insecticidal activity of Xenorhabdus isolates from entomopathogenic nematodes in United Kingdom soil and characterization of the xpt toxin loci. Applied and Environmental Microbiology, 72(9), 5895–5907. 16957209 10.1128/AEM.00217-06 PMC1563616

[mbt270032-bib-0091] Seyfert, C.E. , Müller, A.V. , Walsh, D.J. , Birkelbach, J. , Kany, A.M. , Porten, C. et al. (2023) New genetically engineered derivatives of antibacterial Darobactins underpin their potential for antibiotic development. Journal of Medicinal and Pharmaceutical Chemistry, 66, 16330–16341. 10.1021/acs.jmedchem.3c01660 PMC10726357 38093695

[mbt270032-bib-0092] Seyfert, C.E. , Porten, C. & Müller, R. (2023) Darobactine: eine neue Antibiotikaklasse in Entwicklung. Biospektrum, 29(5), 539–541.

[mbt270032-bib-0093] Seyfert, C.E. , Porten, C. , Yuan, B. , Deckarm, S. , Panter, F. , Bader, C. et al. (2022) Darobactins exhibiting superior antibiotic activity by Cryo‐EM structure guided biosynthetic engineering. Angewandte Chemie, International Edition, 62(2), e202214094. 36308277 10.1002/anie.202214094 PMC10107326

[mbt270032-bib-0094] Shi, Y.‐M. , Hirschmann, M. , Shi, Y.‐N. , Ahmed, S. , Abebew, D. , Tobias, N.J. et al. (2022) Global analysis of biosynthetic gene clusters reveals conserved and unique natural products in entomopathogenic nematode‐symbiotic bacteria. Nature Chemistry, 14, 1–12. 10.1038/s41557-022-00923-2 PMC9177418 35469007

[mbt270032-bib-0095] Shima, K. , Ledig, S. , Loeper, N. , Schiefer, A. , Pfarr, K. , Hoerauf, A. et al. (2018) Effective inhibition of rifampicin‐resistant chlamydia trachomatis by the novel DNA‐dependent RNA polymerase inhibitor corallopyronin a. International Journal of Antimicrobial Agents, 52(4), 523–524. 30092271 10.1016/j.ijantimicag.2018.07.025

[mbt270032-bib-0096] Singh, B.N. (1947) Myxobacteria in soils and composts; their distribution, number and lytic action on bacteria. Journal of General Microbiology, 1(1), 1–10. 20238531 10.1099/00221287-1-1-1

[mbt270032-bib-0097] Sucipto, H. , Pogorevc, D. , Luxenburger, E. , Wenzel, S.C. & Müller, R. (2017) Heterologous production of myxobacterial α‐pyrone antibiotics in *Myxococcus xanthus* . Metabolic Engineering, 44, 160–170. 29030273 10.1016/j.ymben.2017.10.004

[mbt270032-bib-0098] Terlouw, B.R. , Blin, K. , Navarro‐Muñoz, J.C. , Avalon, N.E. , Chevrette, M.G. , Egbert, S. et al. (2023) MIBiG 3.0: a community‐driven effort to annotate experimentally validated biosynthetic gene clusters. Nucleic Acids Research, 51(D1), D603–D610. 36399496 10.1093/nar/gkac1049 PMC9825592

[mbt270032-bib-0099] Testolin, G. , Cirnski, K. , Rox, K. , Prochnow, H. , Fetz, V. , Grandclaudon, C. et al. (2020) Synthetic studies of cystobactamids as antibiotics and bacterial imaging carriers lead to compounds with high in vivo efficacy. Chemical Science, 70, 3–1334. 10.1039/c9sc04769g PMC8148378 34123255

[mbt270032-bib-0100] Thaker, M.N. , Waglechner, N. & Wright, G.D. (2014) Antibiotic resistance–mediated isolation of scaffold‐specific natural product producers. Nature Protocols, 9, 1469 EP. 24874813 10.1038/nprot.2014.093

[mbt270032-bib-0101] Thaker, M.N. , Wang, W. , Spanogiannopoulos, P. , Waglechner, N. , King, A.M. , Medina, R. et al. (2013) Identifying producers of antibacterial compounds by screening for antibiotic resistance. Nature Biotechnology, 31(10), 922–927. 10.1038/nbt.2685 24056948

[mbt270032-bib-0102] van Santen, J.A. , Poynton, E.F. , Iskakova, D. , McMann, E. , Alsup, T.A. , Clark, T.N. et al. (2022) The natural products atlas 2.0: a database of microbially‐derived natural products. Nucleic Acids Research, 50(D1), D1317–D1323. 34718710 10.1093/nar/gkab941 PMC8728154

[mbt270032-bib-0103] Ventola, C.L. (2015) The antibiotic resistance crisis: part 1: causes and threats. P T, 40(4), 277–283. 25859123 PMC4378521

[mbt270032-bib-0104] Waksman, S.A. & Schatz, A. (1945) Streptomycin–origin, nature, and properties*††journal series paper of the Department of Microbiology of the New Jersey agricultural Experiment Station, Rutgers university. Journal of the American Pharmaceutical Association, 34(11), 273–291.

[mbt270032-bib-0105] Walesch, S. , Birkelbach, J. , Jézéquel, G. , Haeckl, F.P.J. , Hegemann, J.D. , Hesterkamp, T. et al. (2022) Fighting antibiotic resistance‐strategies and (pre)clinical developments to find new antibacterials. EMBO Reports, 24, e56033. 36533629 10.15252/embr.202256033 PMC9827564

[mbt270032-bib-0106] Wang, C.‐Y. , Hu, J.‐Q. , Wang, D.‐G. , Li, Y.‐Z. & Wu, C. (2024) Recent advances in discovery and biosynthesis of natural products from myxobacteria: an overview from 2017 to 2023. Natural Product Reports, 41, 905–934. 38390645 10.1039/d3np00062a

[mbt270032-bib-0107] Wang, Z. , Koirala, B. , Hernandez, Y. , Zimmerman, M. & Brady, S.F. (2022) Bioinformatic prospecting and synthesis of a bifunctional lipopeptide antibiotic that evades resistance. Science, 376(6596), 991–996. 35617397 10.1126/science.abn4213 PMC10904332

[mbt270032-bib-0108] Waterfield, N.R. , Bowen, D.J. , Fetherston, J.D. , Perry, R.D. & ffrench‐Constant, R.H. (2001) The tc genes of Photorhabdus: a growing family. Trends in Microbiology, 9(4), 185–191. 11286884 10.1016/s0966-842x(01)01978-3

[mbt270032-bib-0109] Wenski, S.L. , Cimen, H. , Berghaus, N. , Fuchs, S.W. , Hazir, S. & Bode, H.B. (2020) Fabclavine diversity in Xenorhabdus bacteria. Beilstein Journal of Organic Chemistry, 16, 956–965. 32461774 10.3762/bjoc.16.84 PMC7214866

[mbt270032-bib-0110] World Health Organization . (2018) Global Antimicrobial Surveillance System (GLASS) Report 2016‐2017. https://apps.who.int/iris/bitstream/handle/10665/279656/9789241515061‐eng.pdf?ua=1

[mbt270032-bib-0111] Wright, G.D. (2017) Opportunities for natural products in 21st century antibiotic discovery. Natural Product Reports, 34(7), 694–701. 28569300 10.1039/c7np00019g

[mbt270032-bib-0112] Wuisan, Z.G. , Kresna, I.D.M. , Böhringer, N. , Lewis, K. & Schäberle, T.F. (2021) Optimization of heterologous Darobactin a expression and identification of the minimal biosynthetic gene cluster. Metabolic Engineering, 66, 123–136. 33872780 10.1016/j.ymben.2021.04.007

[mbt270032-bib-0113] Zaburannyi, N. , Bunk, B. , Maier, J. , Overmann, J. & Muller, R. (2016) Genome analysis of the fruiting body‐forming Myxobacterium *Chondromyces crocatus* reveals high potential for natural product biosynthesis. Applied and Environmental Microbiology, 82(6), 1945–1957. 26773087 10.1128/AEM.03011-15 PMC4784028

[mbt270032-bib-0114] Zborovsky, L. , Kleebauer, L. , Seidel, M. , Kostenko, A. , Eckardstein, L. , Gombert, F.O. et al. (2021) Improvement of the antimicrobial potency, pharmacokinetic and pharmacodynamic properties of albicidin by incorporation of nitrogen atoms. Chemical Science, 12(43), 14606–14617. 34881013 10.1039/d1sc04019g PMC8580050

[mbt270032-bib-0115] Zhou, Q. , Grundmann, F. , Kaiser, M. , Schiell, M. , Gaudriault, S. , Batzer, A. et al. (2013) Structure and biosynthesis of xenoamicins from entomopathogenic *Xenorhabdus* . Chemistry – A European Journal, 19(49), 16772–16779. 24203528 10.1002/chem.201302481

